# Quetiapine and aripiprazole signal differently to ERK, p90RSK and c-Fos in mouse frontal cortex and striatum: role of the EGF receptor

**DOI:** 10.1186/1471-2202-15-30

**Published:** 2014-02-20

**Authors:** Avril Pereira, Betty Zhang, Peter Malcolm, Anthony Sugiharto-Winarno, Suresh Sundram

**Affiliations:** 1 Department of Molecular Psychopharmacology, The Florey Institute of Neuroscience and Mental Health, The University of Melbourne, Kenneth Myer Building, At Genetics Lane on Royal Parade, Parkville, VIC 3010, Australia; 2 Centre for Neuroscience, The University of Melbourne, Parkville, VIC 3010, Australia; 3 Department of Psychiatry, The University of Melbourne, Parkville, VIC 3010, Australia; 4 Northern Psychiatry Research Centre, The Northern Hospital, Cooper Street, Epping, VIC 3076, Australia

**Keywords:** Antipsychotic drugs, Signaling, ERK, p90RSK, c-Fos, Schizophrenia

## Abstract

**Background:**

Signaling pathways outside dopamine D2 receptor antagonism may govern the variable clinical profile of antipsychotic drugs (APD) in schizophrenia. One postulated mechanism causal to APD action may regulate synaptic plasticity and neuronal connectivity via the extracellular signal-regulated kinase (ERK) cascade that links G-protein coupled receptors (GPCR) and ErbB growth factor signaling, systems disturbed in schizophrenia. This was based upon our finding that the low D2 receptor affinity APD clozapine induced initial down-regulation and delayed epidermal growth factor receptor (EGFR or ErbB1) mediated activation of the cortical and striatal ERK response *in vivo* distinct from olanzapine or haloperidol. Here we map whether the second generation atypical APDs aripiprazole and quetiapine affect the EGFR-ERK pathway and its substrates p90RSK and c-Fos in mouse brain, given their divergent agonist and antagonist properties on dopaminergic transmission, respectively.

**Results:**

In prefrontal cortex, aripiprazole triggered triphasic ERK phosphorylation that was EGFR-independent but had no significant effect in striatum. Conversely quetiapine did not alter cortical ERK signaling but elevated striatal ERK levels in an EGFR-dependent manner. Induction of ERK by aripiprazole did not affect p90RSK signaling but quetiapine decreased RSK phosphorylation within 1-hour of administration. The transcription factor c-Fos by comparison was a direct target of ERK phosphorylation induced by aripiprazole in cortex and quetiapine in striatum with protein levels in temporal alignment with that of ERK.

**Conclusions:**

These data indicate that aripiprazole and quetiapine signal to specific nuclear targets of ERK, which for quetiapine occurs via an EGFR-linked mechanism, possibly indicating involvement of this system in its action.

## Background

Dysfunction of dopamine neurotransmission is considered a central feature of schizophrenia, with antipsychotic drugs (APDs) targeting dopamine D2 (D2) receptors to alleviate positive psychotic symptoms in about one half of patients. However the therapeutic window of D2 receptor blockade (65-78%) within which most APDs achieve optimal clinical utility does not extend to the atypical APD clozapine, despite its superior efficacy in treatment resistant schizophrenia. Sub-threshold levels of D2 receptor blockade exerted by clozapine argues for a mechanism of action not solely reliant on D2 receptor antagonism. In accord with this, we have reported that clozapine signals to the mitogen-activated protein kinase-extracellular signal regulated kinase (MAPK-ERK) cascade via G-protein coupled receptor (GPCR) transactivation of the epidermal growth factor (EGF) receptor (EGFR or ErbB1) [1-3]. This was typified by clozapine induced early inhibition and delayed activation of the ERK response in prefrontal cortex (PFC) and striatum dependent on EGFR signaling *in vitr*o [1] and *in vivo*[2,3] unlike olanzapine or haloperidol. The convergence of the ERK cascade with GPCR and growth factor signaling systems upon activation by APDs is noteworthy since signal transduction from the cell surface to the nucleus can regulate cortical neurogenesis, synaptogenesis and neurotransmitter release, processes affected in schizophrenia [4-6]. Whether these cell signaling effects observed with clozapine extend to the second generation atypical APDs aripiprazole and quetiapine has not been tested. This is relevant given that aripiprazole and quetiapine display agonist and antagonist properties in animal models of dopaminergic hypoactivity and hyperactivity, respectively, share clozapine’s low D2 receptor binding profile (quetiapine) and are predicted to stabilize dopaminergic transmission.

Aripiprazole differs from other atypical APDs in that it acts by partial agonism at D2 and serotonin (5HT) 1A receptors [7]. The drug modulates dopaminergic activity in areas where dopamine may be increased (mesolimbic regions) or diminished (mesocortical regions) in the brains of people with schizophrenia. Like other atypical APDs, aripiprazole antagonizes 5HT2A receptors and has moderate affinity for histamine and α-adrenergic receptors. Quetiapine is a multiple receptor antagonist with low affinity for D2 and higher affinity for 5HT2A, 5HT1A, α-1 and α-2 adrenergic and histamine H1 receptors [8]. Positron emission topography studies indicate that quetiapine rapidly disassociates from the D2 receptor producing normal physiological surges of dopamine in the nigrostriatal and tuberoinfundibular tracts of the brain, thus minimizing the risk of extrapyramidal side effects (EPS) and elevations in prolactin. For both aripiprazole and quetiapine, however, effects on downstream ERK signaling that can regulate transcription factors such as Elk1 or CREB to shape gene expression, protein synthesis and receptor function is less well characterized. In this regard, acute aripiprazole treatment in mice reduced PFC ERK phosphorylation levels in one recorded study to date [9]. By contrast, single time point experiments in Chinese Hamster Ovary (CHO) cells stably transfected with D2 short and 5HT1A [10] and D3 receptors [11] indicated that aripiprazole stimulated ERK phosphorylation via agonist activities at these receptors. Moreover, Urban et al. [12] reported that aripiprazole exerted only partial activation of the ERK pathway in CHO cells expressing D2 long receptors, whereas in PC12 cells aripiprazole promoted neurite outgrowth through activation at 5HT1A rather than D2 receptors and by Ca^2+^, inositol 1,4,5-triphosphate receptor and ERK signaling [13]. Given the cell-dependent differences in the intrinsic activity of aripiprazole, a primary mechanism of action attributable to its functional selectivity at the D2 receptor and/or combined action at non-dopamine receptor systems rather than simple partial agonism has also been argued [12]. Quetiapine too induces ERK mediated neurite sprouting via Gi/o coupled receptors in PC12 cells [14], and activated ERK2 after acute treatment in C6 glioma cells thought to be related to the putative antidepressant efficacy of the drug [15]. In vivo, quetiapine facilitated oligodendrocyte development and prevented cuprizone induced cognitive impairments via ERK in mouse cortex [16] and upregulated ERK gene expression in rat cortex and striatum when administered acutely in conjunction with the mood stabilizer valproate [17]. Whilst cortical and/or striatal ERK phosphorylation by aripiprazole and quetiapine integrate multiple signaling pathways to regulate neuronal processes relevant to the symptom domains of schizophrenia, there remains a paucity of data on the effects of these APDs on the expression of downstream proteins such as 90 kDa ribosomal s6 protein kinase (p90RSK) or c-fos, which potentially define their distinct clinical profiles.

p90RSK comprising the isoforms RSK1, RSK2 and RSK3 are a family of broadly expressed serine/threonine kinases activated by ERK. As a regulator of transcription, p90RSK phosphorylates the transcription factor cyclic AMP response element binding (CREB), which leads to the recruitment of transcriptional co-activators CREB binding protein and the induction of immediate-early genes such as c-Fos [18]. ERK1 knock-out mice exhibit reduced phosphorylation of RSK1 in PFC and striatum, but not in hippocampus or cerebellum indicating ERK signaling deficits that are isoform and region-specific [4]. However there is limited data on the effects of APDs on p90RSK levels, with aripiprazole or quetiapine treatment effects not documented. Similarly there is limited data for aripiprazole and quetiapine in relation to c-Fos which signals a genomic response to a variety of stimuli including growth factors and neurotransmitters, with regulation via the phosphorylation of transcription factors Elk-1 and CREB by ERK and RSK respectively [19]. When compared with other D2 receptor partial agonists, aripiprazole caused less rotation in nigrostriatal lesioned rats (hypo-dopaminergic model) but clear Fos induction in the nucleus accumbens shell, indicative of low intrinsic activity despite functional antagonism, a purported marker of its antipsychotic efficacy [20]. For quetiapine, elevated c-Fos expression in limbic but not motor related brain regions [8,21] with a greater increase in Fos immunoreactivity in rat nucleus accumbens shell than dorsolateral striatum is in keeping with its atypical index and reduced EPS propensity [22]. Apart from these data, the effects of aripiprazole and quetiapine on p90RSK and c-Fos signaling via the ERK pathway and the interrelated EGFR system and how these may differ from clozapine are yet to be profiled.

From this standpoint and to examine whether ERK pathway signaling and transactivation of the EGFR is a mechanism that applies to atypical APDs other than clozapine, the studies undertaken here expand our earlier *in vitro* neuronal culture and *in vivo* animal experiments [1-3] to (i) determine whether acute aripiprazole or quetiapine treatment differentially regulates ERK1/2 phosphorylation over a 24 hr period in mouse PFC or striatum (ii) assess whether changes in ERK1/2 phosphorylation parallel changes in expression of the transcriptional regulators p90RSK and c-Fos across 24 hrs in PFC or striatum and (iii) establish if variations in ERK1/2, p90RSK or c-Fos levels following aripiprazole or quetiapine treatment in cortex or striatum are dependent on EGFR signaling. The PFC and striatum are examined given their relevance to APD signaling and innervation by the major dopamine tracts of the brain, the mesocortical and nigrostriatal pathways, respectively, and also by glutamatergic and gamma-amino-butyric acid (GABA)-ergic neurons [23]. Both regions are important in the pathology of schizophrenia, since the PFC is linked with cognitive, negative and deficit syndrome symptoms and the striatum with motor control, reward and decision making and the EPS triggered by some APDs.

## Results

A summary of all significant pERK1/2, P-p90RSK and c-Fos findings in mouse PFC and striatum following aripiprazole and quetiapine treatment over 24 hrs is provided in Table 1.

**Table 1:** Summary of the significant pERK1, pERK2, P-p90RSK and c-Fos findings in mouse prefrontal cortex and striatum following aripiprazole and quetiapine treatment over 24 hours

**APD**	**Brain region**	**Protein**	**20 min**	**60 min**	**240 min**	**480 min**	**24/24**
Aripiprazole	Prefrontal cortex	pERK1	↓	↑	↓	-	-
		pERK2	↓	↑	-	-	-
		P-p90RSK	-	-	-	-	-
		c-Fos	-	↑	-	-	-
	Striatum	pERK1	-	-	-	-	-
		pERK2	-	-	-	-	-
		P-p90RSK	-	-	-	-	-
		c-Fos	↑	↑	-	-	-
Quetiapine	Prefrontal cortex	pERK1	-	-	-	-	-
		pERK2	-	-	-	-	-
		P-p90RSK	↓	-	-	-	-
		c-Fos	-	-	↑	-	-
	Striatum	pERK1	-	-	↑	-	-
		pERK2	-	-	-	-	-
		P-p90RSK	↓	↓	-	-	-
		c-Fos	-	-	↑	-	-

↓ **=** significantly decreased protein phosphorylation.

↑ **=** significantly increased protein phosphorylation.

- = no significant change.

APD = antipsychotic drug, min = minutes.

### Effect of aripiprazole and quetiapine over 24 hours on ERK phosphorylation in mouse prefrontal cortex and striatum

**Figure 1:**
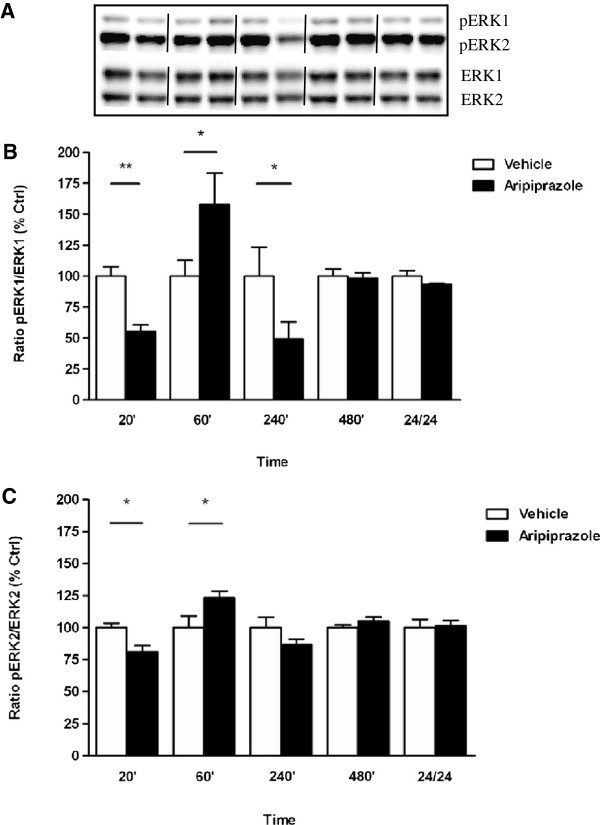
**Effect of aripiprazole on ERK phosphorylation in C57Bl/6 mouse prefrontal cortex.** Representative blots **(A)** indicate immunoreactive bands of phosphorylated ERK1 and phosphorylated ERK2 (upper panel) and total ERK1 and total ERK2 (lower panel) levels following aripiprazole (1 mg/kg) treatment and correspond with the bar graphs below. **(B)** Aripiprazole treatment over a 24 hr period - pERK1/ERK1. **(C)** Aripiprazole treatment over a 24 hr period - pERK2/ERK2. At each time point treated samples were expressed relative to vehicle control standardized to 100 percent. Data represent the mean ± SEM of at least four mice per experimental group. *p < 0.05; **p < 0.01, statistical differences between tissue in the absence (vehicle) and presence of aripiprazole are indicated.

**Figure 2:**
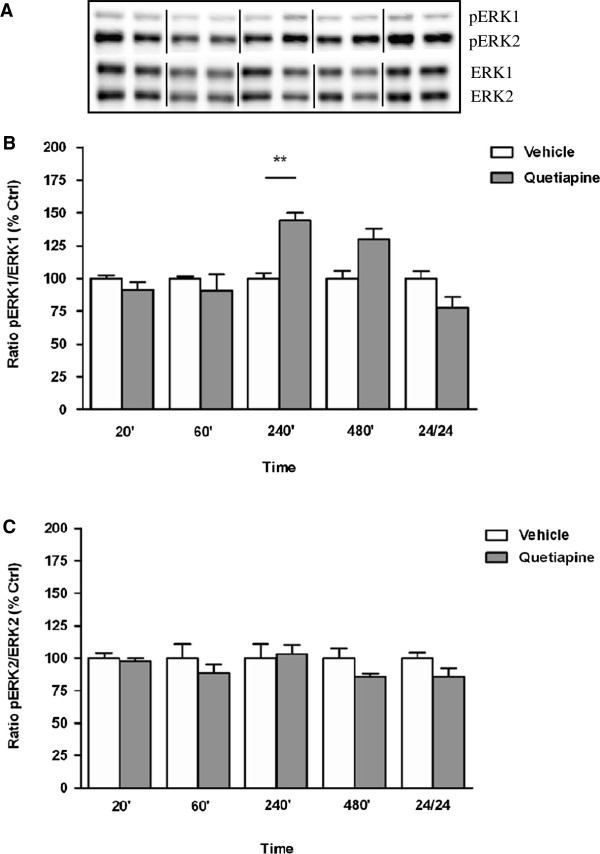
**Effect of quetiapine on ERK phosphorylation in C57Bl/6 mouse striatum.** Representative blots **(A)** indicate immunoreactive bands of phosphorylated ERK1 and phosphorylated ERK2 (upper panel) and total ERK1 and total ERK2 (lower panel) levels following quetiapine (5 mg/kg) treatment and correspond with the bar graphs below. **(B)** Quetiapine treatment over a 24 hr period - pERK1/ERK1. **(C)** Quetiapine treatment over a 24 hr period - pERK2/ERK2. At each time point treated samples were expressed relative to vehicle control standardized to 100 percent. Data represent the mean ± SEM of at least four mice per experimental group. **p < 0.01, statistical differences between tissue in the absence (vehicle) and presence of quetiapine are indicated.

Exposure to aripiprazole resulted in region-specific pERK1/2 findings with phosphorylation altered in the PFC (pERK1: F_(9, 23)_ = 5.606, *p* = 0.0004; pERK2: F_(9, 25)_ = 5.146, *p* = 0.0005) (Figure 1A and B) but not in the striatum (data not shown) Table 1. Triphasic cortical ERK phosphorylation was noted, with pERK1/2 levels decreased at 20 min (pERK1: vehicle 100 ± 8% *vs* aripiprazole 56 ± 5%, *p* < 0.01), increased by 60 min (pERK1: vehicle 100 ± 13% *vs* aripiprazole 158 ± 25%, *p* < 0.05), decreased by 240 min (pERK1: vehicle 100 ± 24% *vs* aripiprazole 49 ± 14%, *p* < 0.05) and normalised thereafter. The significant effects detected for pERK1 were more apparent than those observed for pERK2 (Figure 1A and B). By contrast, quetiapine treatment over a 24 hr period, did not significantly affect ERK1 and ERK2 phosphorylation in mouse PFC (data not shown) (Table 1). In striatum, a marked increase in pERK1 activation was observed only at 240 min (F_(9, 24)_ = 6.930, *p* < 0.0001; vehicle 100 ± 4% *vs* quetiapine 144 ± 6%,1 *p* < 0.01) with levels normalizing by 24 hr (Figure 1A) while pERK2 levels did not vary significantly between untreated and quetiapine treated mice at any time point (Figure 2B).

### Effect of aripiprazole and quetiapine in the absence and presence of AG1478 on ERK phosphorylation in mouse prefrontal cortex and striatum

**Figure 3:**
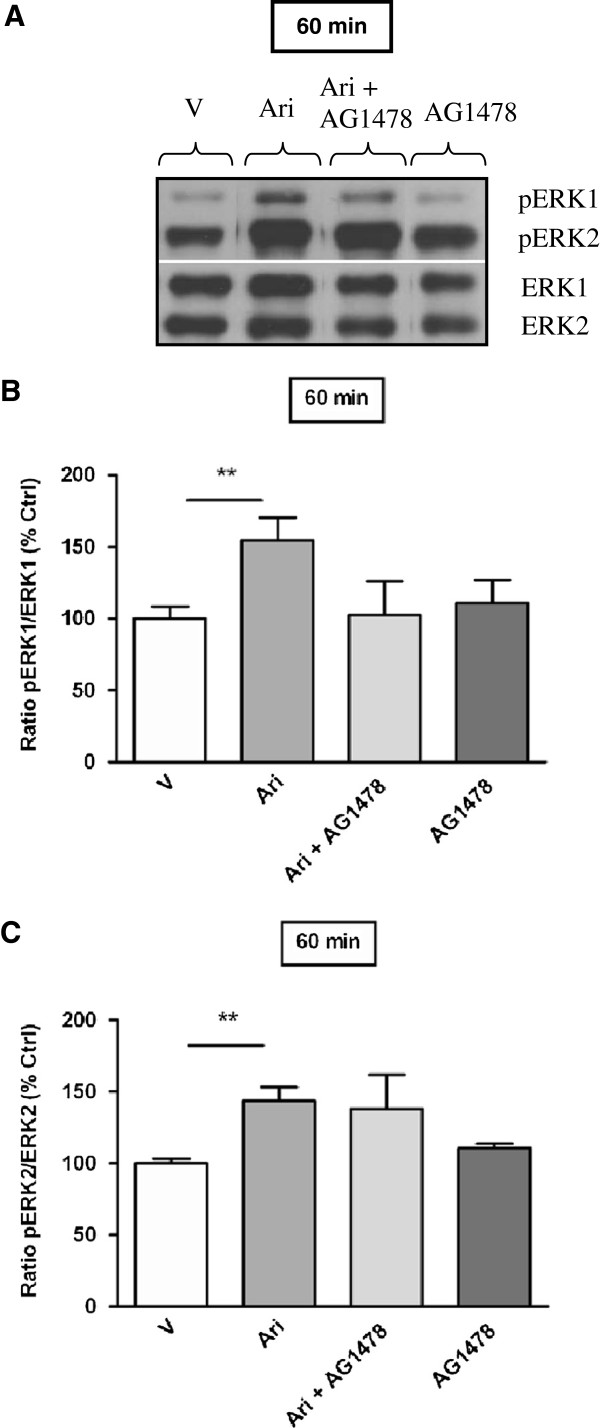
**Effect of aripiprazole on ERK phosphorylation in C57Bl/6 mouse prefrontal cortex in the absence or presence of AG1478 (EGF receptor inhibitor).** Representative blots **(A)** indicate immunoreactive bands of phosphorylated ERK1 and phosphorylated ERK2 (upper panel) and total ERK1 and total ERK2 (lower panel) levels following aripiprazole (1 mg/kg) ± AG1478 treatment and correspond with the bar graphs below. **(B)** Effect of AG1478 on aripiprazole induced ERK1 phosphorylation at 60 min. **(C)** Effect of AG1478 on aripiprazole induced ERK2 phosphorylation at 60 min. Data are expressed relative to vehicle control standardized to 100 percent and represent the mean ± SEM of at least four mice per experimental group. **p < 0.01, statistical differences between tissue in the absence (V) and presence of aripiprazole are indicated. V = Vehicle, Ari = Aripiprazole.

**Figure 4:**
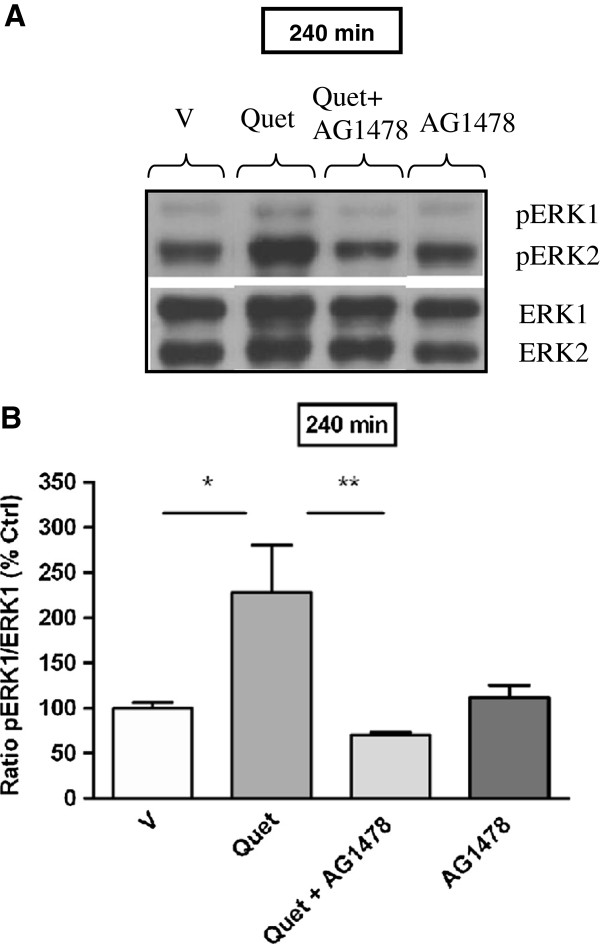
**Effect of quetiapine on ERK1 phosphorylation in C57Bl/6 mouse striatum in the absence or presence of AG1478 (EGF receptor inhibitor) at 240 min.** Representative blots **(A)** indicate immunoreactive bands of phosphorylated ERK (upper panel) and total ERK (lower panel) levels at 240 min following quetiapine (5 mg/kg) ± AG1478 treatment and correspond with the bar graph below. (**B**) Effect of AG1478 on quetiapine induced ERK1 phosphorylation at 240 min. Data are expressed relative to vehicle control standardized to 100 percent and represent the mean ± SEM of at least four mice per experimental group. *p < 0.05; **p < 0.01, statistical differences between tissue in the absence (V) and presence of quetiapine and quetiapine in the absence and presence of AG1478 are indicated. V = Vehicle, Quet = Quetiapine.

For aripiprazole, ERK activation of both isoforms was observed in the PFC after 60 min of drug treatment and hence the effect of AG1478 was examined at this time point. As previously, aripiprazole produced a significant increase in ERK1 and ERK2 phosphorylation in mouse PFC at 60 min (pERK1: 60 min vehicle 100 ± 8% *vs* aripiprazole 155 ± 16%, *p* < 0.01; pERK2: 60 min vehicle 100 ± 3% *vs* aripiprazole 144 ± 10%, *p* < 0.01). However, these effects were not attenuated by AG1478 (Figure 3A, B and C). Contrary to this, quetiapine modulation of striatal ERK1 phosphorylation was significantly reduced by pre-treatment with AG1478 (pERK1: 240 min quetiapine 228 ± 52% *vs* quetiapine + AG1478 70 ± 3%, *p* < 0.01), whereas AG1478 itself did not significantly change pERK1 levels relative to vehicle control (Figure 4A and B).

### Effect of aripiprazole and quetiapine over 24 hours on p90RSK phosphorylation in mouse prefrontal cortex and striatum

**Figure 5:**
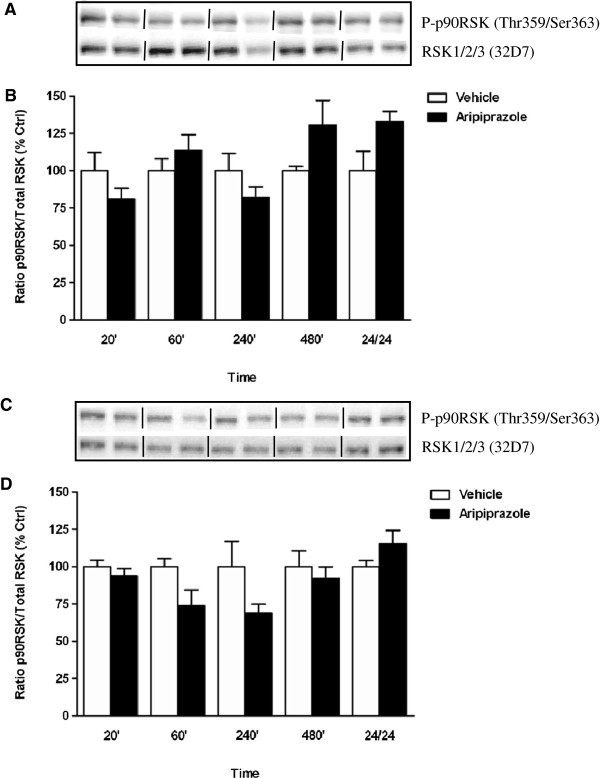
**Effect of aripiprazole on p90RSK phosphorylation in C57Bl/6 mouse prefrontal cortex and striatum.** Representative blots **(A)** prefrontal cortex and **(C)** striatum indicate immunoreactive bands of phospho-p90RSK (upper panel) and RSK1/RSK2/RSK3 (lower panel) following aripiprazole (1 mg/kg) treatment and correspond with the bar graph below. **(B)** Aripiprazole treatment over a 24 hr period - prefrontal cortex. **(D)** Aripiprazole treatment over a 24 hr period - striatum. At each time point treated samples were expressed relative to vehicle control standardized to 100 percent. Data represent the mean ± SEM of at least four mice per experimental group.

**Figure 6:**
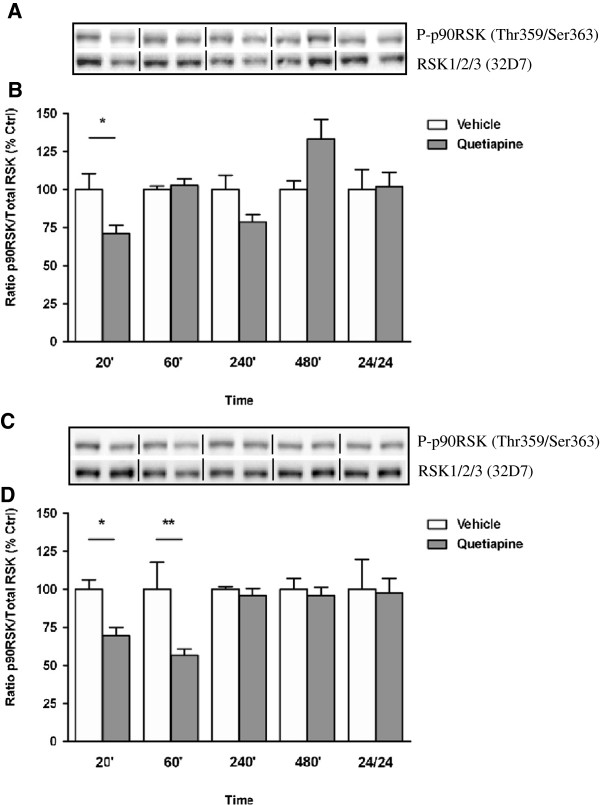
**Effect of quetiapine on p90RSK phosphorylation in C57Bl/6 mouse prefrontal cortex and striatum.** Representative blots **(A)** prefrontal cortex and **(C)** striatum indicate immunoreactive bands of phospho-p90RSK (upper panel) and RSK1/RSK2/RSK3 (lower panel) following quetiapine (5 mg/kg) treatment and correspond with the bar graph below. **(B)** Quetiapine treatment over a 24 hr period - prefrontal cortex. **(D)** Quetiapine treatment over a 24 hr period - striatum. At each time point treated samples were expressed relative to vehicle control standardized to 100 percent. Data represent the mean ± SEM of at least four mice per experimental group. *p < 0.05; **p < 0.01, statistical differences between tissue in the absence (vehicle) and presence of quetiapine are indicated.

Aripiprazole significantly altered p90RSK phosphorylation in the PFC overall compared to vehicle treated animals (F_(9, 23)_ = 3.485, *p* < 0.0075) but elevations in p90RSK phosphorylation at 480 min and 24 hr were not significant after post hoc corrections (Figure 5A). Similarly, aripiprazole administration in mouse striatum produced non-significant changes in p90RSK phosphorylation with decreases at 60 and 240 min failing to reach significance (Figure 5B). Alternatively over a 24 hr period, quetiapine treatment significantly affected p90RSK phosphorylation in mouse PFC (F_(9, 24)_ = 3.648, *p* < 0.0054) and striatum (F_(9, 23)_ = 3.781, *p* < 0.0048) (Figure 6A and B). This was attributable to a significant decrease in p90RSK phosphorylation at 20 min in the PFC (20 min vehicle 100 ± 11% vs quetiapine 71 ± 5%, *p* < 0.05) (Figure 6A) whilst in the striatum, significant reductions at 20 and 60 min (20 min vehicle 100 ± 6% vs quetiapine 69 ± 6%, *p* < 0.05; 60 min vehicle 100 ± 18% vs quetiapine 56 ± 4%, *p* < 0.01) were observed, with levels normalizing thereafter (Figure 6B).

Given that aripiprazole and quetiapine triggered variable patterns of p90RSK phosphorylation in PFC and striatum but produced no significant increases in either region, the effect of AG1478 exposure to these APDs on p90RSK phosphorylation was not examined.

### Effect of aripiprazole and quetiapine over 24 hours on c-Fos expression in mouse prefrontal cortex and striatum

**Figure 7:**
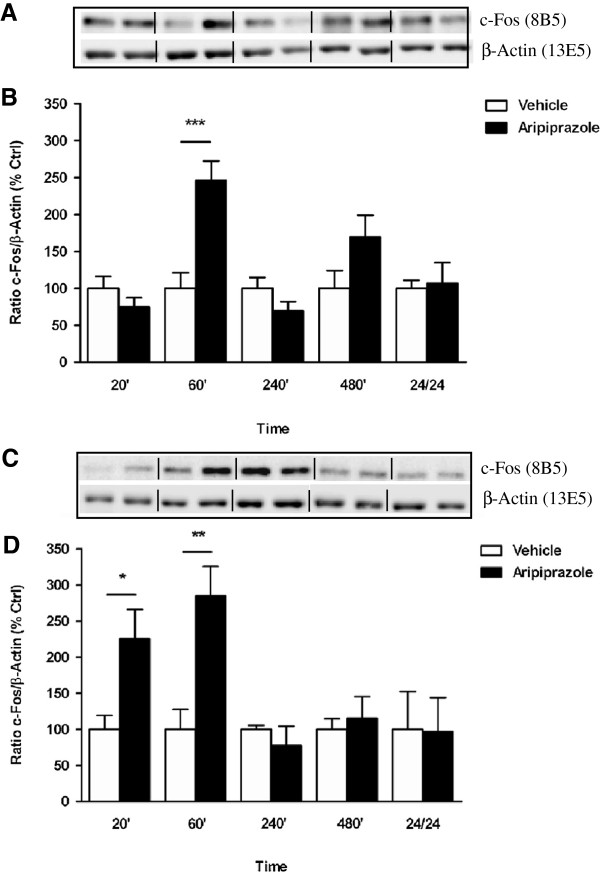
**Effect of aripiprazole on c-Fos expression in C57BL/6 mouse prefrontal cortex and striatum.** Representative blots **(A)** prefrontal cortex and **(C)** striatum indicate immunoreactive bands of c-Fos (upper panel) and β-Actin (lower panel) expression following aripiprazole (1 mg/kg) treatment and correspond with the bar graph below. **(B)** Aripiprazole treatment over a 24 hr period - prefrontal cortex. **(D)** Aripiprazole treatment over a 24 hr period - striatum. At each time point treated samples were expressed relative to vehicle control standardized to 100 percent. Data represent the mean ± SEM of at least four mice per experimental group. *p < 0.05; **p < 0.01; ***p < 0.001, statistical differences between tissue in the absence (vehicle) and presence of aripiprazole are indicated.

**Figure 8:**
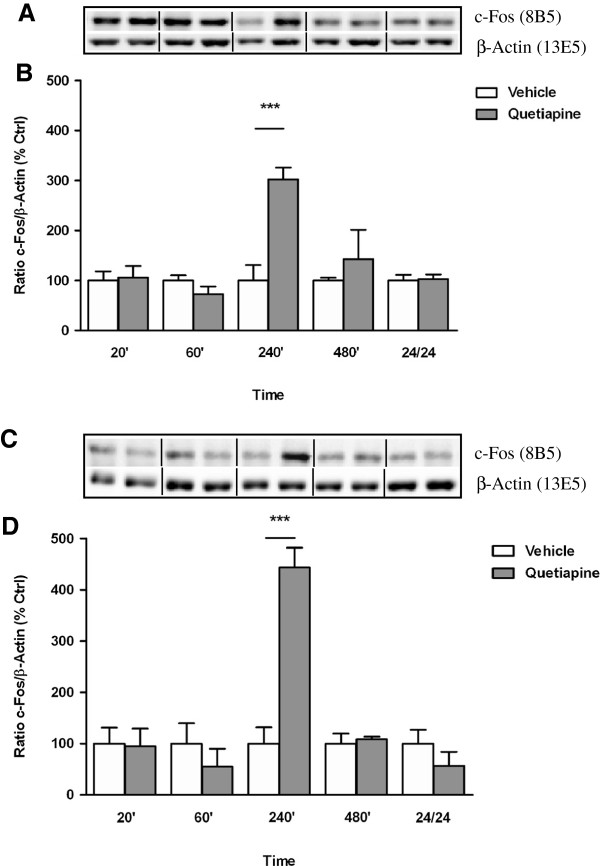
**Effect of quetiapine on c-Fos expression in C57BL/6 mouse prefrontal cortex and striatum.** Representative blots **(A)** prefrontal cortex and **(C)** striatum indicate immunoreactive bands of c-Fos (upper panel) and β-Actin (lower panel) expression following quetiapine (5 mg/kg) treatment and correspond with the bar graph below. **(B)** Quetiapine treatment (5 mg/kg) over a 24 hr period - prefrontal cortex. **(D)** Quetiapine treatment (5 mg/kg) over a 24 hr period - striatum. At each time point treated samples were expressed relative to vehicle control standardized to 100 percent. Data represent the mean ± SEM of at least four mice per experimental group. ***p < 0.001, statistical differences between tissue in the absence (vehicle) and presence of quetiapine are indicated.

Characterization of c-Fos expression in response to aripiprazole in the cortex indicated a significant effect of treatment over the 24 hr period (F_(9, 25)_ = 6.616, *p* < 0.0001) attributed to an increase at 60 min (vehicle 100 ± 21% *vs* aripiprazole 246 ± 26, *p* < 0.001) (Figure 7A). Fluctuations in c-Fos levels in the striatum after aripiprazole treatment also reached significance (F_(9, 22)_ = 3.420, *p* = 0.0089) with elevations seen at 20 min (vehicle 100 ± 19% *vs* aripiprazole 226 ± 40, *p* < 0.05) and 60 min (vehicle 100 ± 27% *vs* aripiprazole 285 ± 41, *p* < 0.01) (Figure 7B). Following quetiapine administration, a marked increase in c-Fos protein was observed at 240 min in PFC (vehicle 100 ± 31% *vs* quetiapine 302 ± 24, *p* < 0.001) and striatum (vehicle 100 ± 32% *vs* quetiapine 444 ± 39, *p* < 0.001) with levels comparable to vehicle treatment at other times across 24 hr (Figure 8A and B, respectively).

### Effect of aripiprazole and quetiapine in the absence and presence of AG1478 on c-Fos expression in mouse prefrontal cortex and striatum

The increased c-Fos levels caused by aripiprazole at 60 min in PFC and striatum were not significantly affected by prior administration of AG1478 in either region (PFC: aripiprazole 100 ± 13% *vs* aripiprazole + AG1478 140 ± 10%; striatum: aripiprazole 100 ± 1% *vs* aripiprazole + AG1478 85 ± 2%, *p* > 0.05 in both cases). Similarly for quetiapine, elevations in c-Fos expression observed at 240 min in cortex and striatum were not significantly reduced by AG1478 (PFC: quetiapine 100 ± 3% *vs* quetiapine + AG1478 75 ± 10%; striatum: quetiapine 100 ± 5% *vs* quetiapine + AG1478 82 ± 26%, *p* > 0.05 in both cases).

## Discussion

### Aripiprazole and quetiapine differentially regulate ERK phosphorylation

The mechanisms underlying the action of aripiprazole and quetiapine on the ERK transduction pathway in PFC and striatum *in vivo* are largely unknown. We therefore determined that aripiprazole triggered triphasic ERK phosphorylation with pERK1 and pERK2 levels first decreased in mouse PFC at 20 min, increased by 60 min, decreased by 4 hrs and normalised thereafter. No striatal pERK changes were noted with aripiprazole treatment. By contrast quetiapine caused no significant changes in ERK1/2 phosphorylation in cortex, while in striatum pERK1 activation was only observed at 240 min. Moreover, cortical ERK induction by aripiprazole was independent of EGFR activity whereas striatal ERK induction by quetiapine was EGFR dependent. The observed differences in pERK1/2 levels were not due to corresponding variation in total ERK1/2 pools since these remained relatively stable across experiments. Rather, significant variability between pERK isoform levels within brain regions at the times tested may suggest that pERK1/2 pools are functionally discrete. Therefore ERK signaling was affected differently by aripiprazole and quetiapine in a time-dependent and region-specific manner and was reliant on EGFR transactivation in the case of quetiapine.

The ERK profile induced by aripiprazole may be a consequence of the drug’s novel receptor binding properties that primarily modulate and stabilize the dopamine system [7,12]. The efficacy of aripiprazole is proposed to be mediated through a combination of partial agonist activity at D2 and 5HT1A receptors and antagonist activity at 5HT2A receptors [24]. Although the functional significance of the cortical pERK1/2 fluctuations observed following aripiprazole administration is not known, they are noteworthy in light of aripiprazole’s clinical effects. For example, the relatively low risk of EPS associated with aripiprazole use [25] is presumed to be due to the drug’s lack of effect on the nigrostriatal pathway of the brain. The latter is in line with our own *in vivo* data indicating an absence of significant changes in striatal pERK1/2 levels after aripiprazole treatment over the time course studied. Our cortical finding of early reduction in ERK1/2 phosphorylation at 20 min also concurs with a single *in vivo* study that recorded a decrease in pERK1/2 levels 15 min after acute aripiprazole administration, although subsequent time points were not examined [9]. Drawing valid comparisons between *in vitro* experiments utilizing D2, D3 and 5HT1A receptor-transfected CHO and PC12 cells exposed to aripiprazole for 5 and 10 min [10-13] and our own *in vivo* work is however difficult given the inherent differences in the biological systems and time frames investigated.

The lack of effect of *in vivo* treatment with quetiapine in cortex contrasts with the ERK phosphorylation profile induced by clozapine [1,2], even though both atypical APDs are D2, 5HT2A and α-1 adrenergic receptor antagonists. Differences in factors such as proportional drug receptor occupancy and affinity state may account for the distinctive ERK signaling response of each APD [26]. For quetiapine, a dose of 5 mg/kg was chosen to limit the sedative properties of the drug that are presumed to occur via histamine H1 receptor antagonist activity [16,27]. Therefore sedation is unlikely to be the reason for the absence of cortical pERK1/2 effects observed. In striatum, quetiapine increased pERK1 activation only at 240 min, unlike the sustained ERK1 phosphorylation previously seen with haloperidol [2]. This is interesting given that quetiapine use is associated with risk of tardive dyskinesia [28] but to a less extent than typical APDs [29]. This reduced risk of motor side-effects is thought to be due to quetiapine’s rapid dissociation from D2 receptors in the nigrostriatal pathway of the brain [30]. However inferences on the clinical effects of quetiapine attributable to ERK activation are limited since they can only be drawn from a few *in vitro* cell studies [14,15], a chronic *in vivo* mouse study [16] and an acute rat gene expression study [17]. In this regard, quetiapine promoted ERK mediated neurite sprouting in PC12 cells [14] with improvements in affect and mood also ascribed to ERK signaling [15,17], and in mouse cortex prevented cuprizone induced myelin breakdown and cognitive impairments via ERK dependent on EGF [16]. Our data support the latter study insofar as we show an interaction between quetiapine and the EGFR in mediating ERK activation in the striatum.

### Aripiprazole and quetiapine effects on the downstream ERK targets p90RSK and c-Fos

Aripiprazole and quetiapine induced p90RSK phosphorylation did not parallel that of ERK unlike for clozapine [3]. Overall we found that quetiapine decreased p90RSK levels within 1 hr of administration regardless of its concomitant effect on ERK, while aripiprazole did not appreciably affect p90RSK phosphorylation. These data therefore suggest that in PFC and striatum p90RSK is unlikely a major downstream target of ERK signaling in response to aripiprazole or quetiapine and that these drugs differ from clozapine in the way in which they influence distal transcriptional measures [3]. In terms of c-Fos expression however, significant induction at 60 min by aripiprazole and at 240 min by quetiapine corresponded with increased ERK phosphorylation at the same time points in PFC and striatum, respectively. The absence of a time delay between ERK activation and stimulation of c-Fos by aripiprazole and quetiapine again contrasts with our clozapine findings in which c-Fos was expressed subsequent to increases in ERK phosphorylation [3]. For both drugs, elevations in c-Fos were not affected by EGFR inhibition. Thus given that quetiapine induced c-Fos activation in striatum was not affected by AG1478 but ERK phosphorylation was significantly reduced suggests that using a pharmacological agent to block the EGF receptor, an upstream component of the pathway, may inadequately affect a downstream nuclear response. Alternatively, c-Fos activation may have occurred independently of EGFR-ERK pathway induction via other signaling mechanisms or extracellular mitogenic stimuli upon *in vivo* exposure to quetiapine.

For aripiprazole, while c-Fos protein induction in the PFC has not been previously documented, early 20 min striatal (ventral and dorsal) up-regulation observed concurs with high Fos expression in the nucleus accumbens shell (ventral striatum) versus lower levels in the dorsolateral striatum seen 2 hr after acute administration in rat and recognized as markers of antipsychotic efficacy and motor side effects, respectively [20,31]. Similarly for quetiapine, increased c-Fos expression in PFC and striatum at 240 min is in general accordance with acute [22] and chronic [21] data in rat, where Fos-like immunoreactivity was elevated in PFC and ventral striatum although the temporal link to ERK signaling in striatum has not been reported before. While it is understood that APDs do not reach complete effectiveness after a single dose, the present findings are of mechanistic value and allow future studies to be designed to examine whether there is any difference in signaling induced by sustained treatment with these agents. Moreover, the current studies utilizing mouse neuronal tissue to examine the signaling and hence phosphorylation status of proteins relevant to APD action cannot be undertaken in human subjects (with schizophrenia). Therefore the animal experiments described permit some delineation of the specific intracellular pathways targeted by APDs. In doing so, these studies may point to potential candidate proteins and mechanisms affected by APDs and may shed light on psychotic disorders such as schizophrenia.

### Antipsychotic drugs and the EGF receptor system

We had earlier proposed that clozapine may be unique in recruiting the EGFR system to target ERK and that this may have some bearing on clozapine’s unequaled ability to treat drug resistant schizophrenia [1-3]. Here in keeping with our hypothesis ERK induction by aripiprazole was EGFR independent but contrary to our supposition, quetiapine induced ERK activation was EGFR dependent. There are however important differences between quetiapine and clozapine signaling including: i) Regional differences in ERK expression. Quetiapine activated ERK in striatum only and had no effect in the PFC whereas clozapine was able to induce ERK in mouse PFC and striatum. ii) Temporal differences in ERK expression. Quetiapine affected striatal ERK phosphorylation at 240 min but not at other time points across the treatment schedule. By contrast, clozapine inhibited ERK phosphorylation within 1 hr of administration, and then activated ERK at 480 min in both brain regions. Therefore, while quetiapine and clozapine share the ability to recruit the EGFR to signal to ERK, they nevertheless control ERK phosphorylation differently. Notably, quetiapine is not effective in treatment resistant schizophrenia unlike clozapine. Whether differences in ERK expression provide additional benefits for clozapine-treated patients that may account for the drug’s greater efficacy over other APD remains to be addressed.

The regulation of the EGFR by quetiapine and clozapine is a novel mechanism of APD action with potential implications for the treatment of schizophrenia. Besides playing a central role in the development of midbrain dopaminergic neurons, impaired EGF system functioning has been linked to the pathogenesis of the disorder. For example, genetic association studies have identified the EGF and NRG1 genes as candidates that confer risk for schizophrenia [32] with an A61G single nucleotide polymorphism in the EGF gene associated with early disease onset in male patients [33]. As well in human post-mortem brain in schizophrenia, increased EGFR density was considered to offset the low EGF levels noted [34]. Furthermore while perturbation to neonatal EGF signaling resulted in behavioural brain abnormalities in adulthood consistent with those present in animal models of schizophrenia [35], the EGF family of ligands and receptors also affect neuronal growth, differentiation and survival later in development [36]. Thus given that several studies argue for EGF system disturbance in schizophrenia, the current findings indicate a possible corrective role for APDs such as quetiapine.

Just which GPCR is employed by quetiapine or clozapine to undertake EGFR transactivation is unclear. Both APDs share the property of being D2/5HT2A antagonists. While we have demonstrated in primary mouse cortical neurons that clozapine induced transactivation of the EGFR occurred independently of the D2 or 5HT2A receptor [1], whether this holds true for quetiapine is unclear. The transactivation pathway that signals to the EGFR also needs to be elucidated for quetiapine and clozapine, bearing in mind that there may be regional differences.

## Conclusions

These *in vivo* studies highlight that the atypical APDs aripiprazole and quetiapine exert unique temporal and regional regulation of the convergent EGFR-ERK pathway and its downstream transcriptional targets, p90RSK and c-Fos in PFC and striatum that may account for their distinctive clinical profiles. In this regard, cortical pERK1 stimulation by aripiprazole was EGFR independent whereas striatal pERK1 activation by quetiapine was EGFR dependent. While aripiprazole induction of ERK had no significant specific effect on p90RSK signaling, quetiapine reduced RSK phosphorylation early after exposure. By contrast, c-Fos expression induced by aripiprazole in cortex and quetiapine in striatum temporally aligned with ERK phosphorylation and was indicative of transcriptional regulation as a direct corollary of APD induced ERK signaling. Collectively, these data provide further evidence that APD action via ERK may be linked to the EGF signaling system, perturbations of which have been documented in schizophrenia and suggest a remedial role for APDs such as quetiapine.

## Methods

### Drugs and reagents

All reagents were obtained from Sigma-Aldrich, Missouri, USA unless stipulated otherwise. Quetiapine was donated by AstraZeneca, Stockholm, Sweden; aripiprazole by Bristol-Myers Squibb, New Jersey, USA and AG1478 (EGFR inhibitor) purchased from A.G. Scientific, Inc., California, USA. Primary antibodies, including phospho-p44/42 MAPK, p44/42 MAP kinase, phospho-p90RSK, RSK1/RSK2/RSK3 and β-Actin were from Cell Signaling Technology, Massachusetts, USA and c-Fos from Assay Designs, Michigan, USA. Secondary antibodies, including goat anti-mouse and goat anti-rabbit horseradish peroxidase (HRP)-conjugated immunoglobulins (IgGs) were supplied by DAKO, NSW, Australia.

### Animals

Animal care and experimental procedures were conducted in accordance with The University of Melbourne Animal Ethics Committee guidelines. Male, 7 week old C57Bl/6 mice were housed under standard laboratory conditions on a 12-hour light–dark cycle (lights on 07:00 hr) and provided free access to food and water. Animals were habituated to the laboratory facility for one week; were handled daily to reduce acute stress and were weighed before drug treatment.

### Aripiprazole and quetiapine time course studies

For acute time course experiments, groups of mice (n = 4) were treated via intraperitoneal (IP) injection with the APDs aripiprazole (1 mg/kg) or quetiapine (5 mg/kg) dissolved in 0.9% saline acidified with 0.1 N HCl or vehicle (1% v/v) as a single dose and were left for 20, 60, 240, 480 min or 24 hr after administration. The doses injected were in the mid-range of those used in mouse studies and in line with APD dose in humans. Such doses were also known to cause effects consistent with antipsychotic mouse models of psychosis without sedation [1,27,37,38]. Directly following the time interval specified and in order to preserve phospho-proteins, mice were decapitated, the head immersed in liquid nitrogen for 6 sec, the brain rapidly removed and PFC and striatum dissected out within 20 sec on an ice-cold platform [2,3]. Brain tissue was sonicated in 1% SDS (750 μl), boiled for 10 min and frozen at -80°C until assayed. Before protein determination, lysates were centrifuged at 14000 × *g* for 5 min at 4°C to remove insoluble material. Lysate protein content was measured by the Bradford method (Bio-Rad Protein Assay, California, USA) using BSA as standard. Brain lysates were assayed for phosphorylated and total ERK1, ERK2, p90RSK and c-Fos levels as outlined.

### AG1478 treatment studies

To determine the effect of EGFR inhibition on ERK phosphorylation and c-Fos expression, mice (n = 4 per group) were treated with AG1478 (EGFR inhibitor) at 25 mg/kg dissolved in 50% DMSO 10 min prior to APD or vehicle administration. In the case of aripiprazole and quetiapine, co-treatment with AG1478 was performed at 60 and 240 min, respectively, time points at which each drug had significantly activated ERK and c-Fos above vehicle. For experiments that spanned 240 min, two injections of AG1478 were given 2 hr apart to maintain adequate plasma levels [39]. At the experimental endpoint, PFC and striatal tissue was dissected out as described.

### ERK1/2, p90RSK and c-Fos assay

Aliquots of 15–30 *μ*g of PFC and striatal protein lysate were separated by SDS-PAGE and immunoblotted using standard methods. Proteins were electrotransferred to nitrocellulose membrane (Osmonics, Minnesota, USA) and blocked at room temperature for 90 min in 5% skim milk powder, TBST (20 mM Tris-Base pH 7.5, 150 mM NaCl, 0.01% Tween-20). Membranes were incubated overnight with primary phospho-p44/42 MAP Kinase (Thr202/Tyr204) (E10) antibody (1:2000) in blocking buffer or phospho-p90RSK (Thr359/Ser363) antibody (1:1000) in 5% BSA and secondary goat anti-mouse and goat anti-rabbit HRP-conjugated IgGs (1:2000), respectively, in blocking buffer for 90 min at 4°C. c-Fos levels were assessed using c-Fos (8B5) mouse monoclonal antibody (1:500) and goat anti-mouse HRP-conjugated IgGs (1:2000) in blocking buffer. Following primary and secondary antibody exposure, membranes were washed twice in TBST for 15 min at room temperature. Immunoreactive bands were detected using ECL Western Blotting Detection Reagents, (Amersham Biosciences, Buckinghamshire, UK) and Hyperfilm ECL (Amersham Biosciences). To ensure uniform loading, membranes were stripped in 62.5 mM Tris–HCl at pH 6.7, 2% SDS and 100 mM β-mercaptoethanol buffer at 50°C for 30 min and re-probed with p44/42 MAP Kinase antibody (1:1000) in 5% skim milk, TBST or RSK1/RSK2/RSK3 (32D7) rabbit monoclonal antibody (1:1000) in 5% BSA and goat anti-rabbit HRP-conjugated IgGs (1:2000) for measurement of total ERK1, ERK2 and RSK levels, respectively. Since c-Fos (8B5) detected endogenous levels of total c-Fos protein, β-Actin (13E5) rabbit monoclonal antibody (1:2000) in 5% BSA was used as a loading control. Proteins were quantified using Multi Gauge Software (Fujifilm V3.0). The optical densities of phosphorylated ERK1 (pERK1), phosphorylated ERK2 (pERK2), phosphorylated p90RSK (P-p90RSK) or c-Fos immunoreactive bands were measured, normalized to the optical densities of total ERK1 (ERK1), total ERK2 (ERK2), total RSK and β-Actin, respectively, and expressed as a percentage of vehicle treated control.

### Data analysis

Animal data was pooled with each treatment group repeated in quadruplicate and the mean ± standard error of the mean (SEM) calculated using GraphPad Prism 5 software (GraphPad Software Inc., California, USA). Variables were assessed using one-way analysis of variance (ANOVA) and corrected by post hoc Bonferroni multiple comparison tests to discriminate differences between control and treated groups. Unpaired Student’s (2-tailed) *t*-tests for comparison between pairs of variables were used as appropriate.

## Abbreviations

ANOVA: Analysis of variance; APD: Antipsychotic drugs; BSA: Bovine serum albumin; CHO: Chinese hamster ovary; CREB: Cyclic AMP response element binding; D2: Dopamine D2 receptors; ECL: Enhanced chemiluminescence; EGF: Epidermal growth factor; EGFR or ErbB1: Epidermal growth factor receptor; ERK: Extracellular signal-regulated kinase; ERK1: Total ERK1; ERK2: Total ERK2; GPCR: G-protein coupled receptors; HRP: Horseradish peroxidise; 5HT: Serotonin; IgGs: Immunoglobulins; MAPK: Mitogen activated protein kinase; NRG1: Neuregulin 1; pERK1: Phosphorylated ERK1; pERK2: Phosphorylated ERK2; PFC: prefrontal cortex; P-p90RSK: phosphorylated p90RSK; p90RSK: 90 kDa ribosomal s6 protein kinase; SDS: Sodium dodecyl sulphate; SEM: Standard error of the mean; TBST: Tris buffered saline tween.

## Competing interests

The authors declare that they have no competing interests.

## Authors’ contributions

AP provided intellectual input and oversaw all aspects of study design and implementation; participated in the animal treatment studies; performed Western procedures and data analysis; interpreted data and wrote the manuscript. BZ performed Western procedures and collated, analysed and interpreted data as part of her Advanced Medical Science thesis. PM assisted with the animal treatment studies, performed Western procedures and data analysis. ASW injected animals over time course experiments; performed Western and data analysis. SS provided intellectual input in study design and manuscript preparation; executed procedures in the animal treatment studies and oversaw the work. All authors read and approved the final manuscript.
